# Ropivacaine represses the proliferation, invasion, and migration of glioblastoma via modulating the microRNA-21-5p/KAT8 regulatory NSL complex subunit 2 axis

**DOI:** 10.1080/21655979.2022.2037955

**Published:** 2022-02-22

**Authors:** Zexiang Deng, Yanping Jian, Hongwei Cai

**Affiliations:** Department of Anesthesiology, Xiangya Hospital of Central South University, Changsha City, Hunan Province, China

**Keywords:** Glioblastoma, Ropivacaine, MicroRNA-21-5p, KAT8 regulatory NSL complex subunit 2

## Abstract

Ropivacaine (Rop) is available to suppress the growth of glioblastoma (GBM), while its mechanism has not been completely elaborated. In this study, we explore the latent mechanism of Rop repressing GBM’s growth via mediating the microRNA (miR)-21-5p/KAT8 regulatory NSL complex subunit 2 (KANSL2) axis. MiR-21-5p was declined in GBM, while KANSL2 was elevated. Clinical association studies manifested miR-21-5p was distinctly linked to the tumor size and grade of GBM. Rop constrained GBM cell proliferation, invasion, and migration but boosted apoptosis. Elevated miR-21-5p strengthened Rop’s action, while augmented KANSL2 weakened Rop’s role. Furthermore, the impact of silencing miR-21-5p on GBM was turned around via declining KANSL2 in Rop-treated GBM cells. KANSL2 was the target gene of miR-21-5p. In short, Rop exerted an anti-tumor impact on GBM via mediating the miR-21-5p/KANSL2 axis, which offered novel viewpoints for the later adoption of Rop as GBM drugs.

## Graphical Abstract

**Figure:**
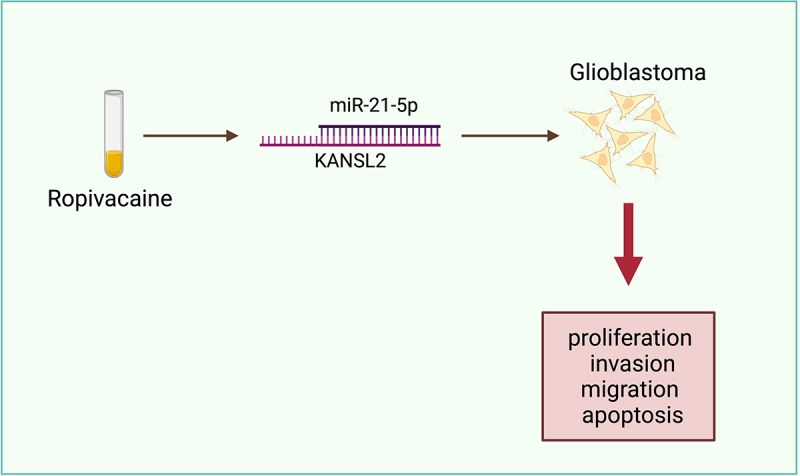

## 1 Introduction

Glioblastoma (GBM) is a popular and extremely aggressive primary malignant tumor in the brain [1,2]. Approximately 52% brain tumors are GBM [3]. The molecular mechanism of the pathogenesis of GBM has not yet been completely figured out. The present treatment for GBM crucially counts on surgery [4], chemotherapy [5] radiotherapy [6], and electric field therapy [7]. These measures are adopted to rescue the lives of patients, but the prognosis of GBM is still unpleasing and the majority of patients relapse after resection [8]. As reported, approximately 90% patients diagnosed with GBM will die within 3 years [9]. Consequently, it is crucial to further explore the pathogenesis of GBM and seek more imperative treatment methods.

Ropivacaine (Rop), a long-acting amide levorotatory local anesthetic, has been broadly adopted in anesthesia or local analgesia [10]. Recently, multiple researches have reported diversified local anesthetics like bupivacaine, Rop, etc. [11]. Manifest remarkable effects in the cure of cancer, mainly presenting in restraining cancer proliferation [12], invasion [13], migrate [14] and transfer [15], etc. The present study elucidated how Rop constrains the growth and self-renewal of GBM stem cells via repressing zinc finger Asp-His-His-Cys-type palmitoyltransferase 15 (ZDHHC15)-mediated palmitoylation of GP130 [16]. Nevertheless, the latent molecular mechanism of Rop repressing GBM remains to be further figured out.

MicroRNA (miRNA), an endogenous non-coding RNA has been discovered to exert a tremendous action in modulating cancer cell proliferation [17], migration [18], apoptosis [19]. MiR-21-5p, belonging to the miRNA family, has been testified to be aberrant in multiple cancers covering colon cancer [20], lung cancer [21], stomach cancer [22] and ovarian cancer [23]. Foregoing researches have elucidated miR-21-5p is declined in GBM, and elevated miR-21-5p is available to repress migration and invasion of GBM and boost apoptosis in *vitro* [24]. Additionally, declined miR-21-5p were also discovered in GBM external vesicles [25]. Nevertheless, the downstream target genes of miR-21-5p modulating GBM’s growth has hardly been reported, and its action on the GBM should be further explored.

In this research, the latent molecular mechanism of Rop suppressing GBM’s growth was explored. Furthermore, the miR-21-5p/KAT8 regulatory NSL complex subunit 2 (KANSL2) pathway is the crucial approach for Rop to constrain GBM.

## 2 Materials and methods

### 2.1 Clinical samples

From 2015 to 2018, a collection of 49 pairs of tumor and para-cancerous normal tissues was from patients undergoing GBM resection at Xiangya Hospital of Central South University. Grading of GBM was in the light of the pathological diagnostic criteria of World Health Organization (WHO). No recipient of the patient had any local or systemic treatment prior to surgery. This research accorded with the regulations of the Ministry of Health of the People’s Republic of China and Declaration of Helsinki on Ethical Principles of Medical Research Involving the Human Body. All patients offered informed consent on the grounds of the plan of the Ethics Committee of Xiangya Hospital of Central South University. Collections of all tissue samples and freeze in liquid nitrogen were immediately performed, and storing was conducted until RNA was extracted.

### 2.2 Cell culture

GBM U87, U373, and U251 cells line and human astrocyte nano-hydroxyapatite (NHA) cell lines were applied (all Cell resource center, SIBS, CAS, Shanghai, China). The culture of the cells was in Roswell Park Memorial Institute 1640 medium (Thermo Fisher Scientific, MA, USA) covering 10% fetal bovine serum (FBS) (Thermo Fisher Scientific) and 1% penicillin/streptomycin (Invitrogen, CA, USA). Detachment of the cells in the logarithmic growth phase was with 0.25% trypsin (Thermo Fisher HyClone, Utah, USA) for cell passage and further experiments. In order to determine the optimal inhibitory concentration of Rop on GBM cell lines, GBM cell lines were treated with different concentrations of Rop (0.5, 1, 5, 10 μmol/L) for 12, 24, 48 h, and cell viability was detected by CCK-8 [26].

### 2.3 Cell grouping

Division of the cells was into the following groups, in which introduction of cells was with 5 μmol/L Rop and/or transfection with the corresponding plasmids except for the mimic-NC, the miR-21-5p-mimic, and the Control. Treatment of cells in the control was with normal saline. There were the Rop, the Rop + mimic-negative control (NC) (mimic-NC), the Rop + miR-21-5p-mimic (miR-21-5p-mimic), the Rop + oe-NC/KANSL2 (oe-NC/KANSL2), the Rop + miR-21-5p-inhibitor + si-NC/KANSL2 (miR-21-5p-inhibitor and si-NC/KANSL2), the mimic-NC (mimic-NC) and the miR-21-5p-mimic (miR-21-5p). Purchase of Rop was implemented (siGBMa, Shanghai, China). Synthesis of miR-21-5p-mimic/inhibitor and the corresponding NCs was conducted (Sangon Biotechnology, Shanghai, China). Purchase of siRNA targeting KANSL2, elevated plasmid, and corresponding NC was conducted (Genepharma, Shanghai, China).

### 2.4 Cell counting Kit-8 (CCK-8)

CCK-8 was performed as previously described [26]. Detachment of U87, U373, and U251 cells was with trypsin, and adjustment was to the cell density of 2 × 10^3^/mL, and then seeding was in a 96-well plate with 100 μL cell suspension per well. After that, placing of the 96-well plate was in the incubator for further culture. After 0, 24, 48, and 72 h, addition of 10 μL CCK-8 solution (Hubei Biotechnology Co., Ltd.) was to each well, and the culture of the cells was in the incubator. Subsequently, placing of the 96-well plate was used in a microplate reader to measure the absorbance at a wavelength of 450 nm.

### 2.5 Flow cytometry test of cell apoptosis

Detection of cell apoptosis was via adopting Annexin V-Fluorescein Isothiocyanate (FITC)/Propidium Iodide (PI) Apoptosis Detection Kit (Shanghai, China) in the light of the manufacturer’s instructions [27]. After 48 h of incubation, the rinse of U87, U373, and U251 cells was with PBS, the resuspension was in the binding buffer, and then the double staining was with Annexin V-FITC and PI. Analysis of the apoptosis rate was via adopting the flow cytometer (Becton-Dickinson, Franklin Lakes, NJ, USA) and CellQuest software (BD Biosciences).

### 2.6 Transwell examination of cell invasion

The seeding of the transfected cells was into the chamber of Transwell (2.5 × 10^4^ cells/well), and resuspension of the cells in the chamber was in serum-free medium, while the bottom of the chamber was medium covering 20% serum. Pre-coating of the matrigel layer is placed into the inner chamber to simulate the extracellular matrix [28]. After incubation, taking of the cells was from the chamber, and fixation of the cells in the lower wells was via the mixture of formaldehyde and acetic acid, and staining was with crystal violet. Ultimately, a count of the number of invaded cells was conducted.

### 2.7 Scratch test detection of cell migration

The electroblot of the cells (2 × 10^5^/well) was onto a 12-well plate, and the culture was in Dulbecco’s Modified Eagle Medium with 10% FBS. Once the confluence of the cells reached 100%, scratch of the monolayer cells was with a 200 mL tip, and rinse of the wells was with serum-free medium for 3 times; Observation of the wound was conducted and pictures were taken. Then culture of the cells was in serum-free medium [29]. After 24 h, observation of the wound and pictures was taken again. Scratch healing rate = 100%-(0 h scratch width-24 h scratch width)/0 h scratch width × 100%.

### 2.8 Reverse transcription quantitative polymerase chain reaction (RT-qPCR)

Extraction of RNA was from tissues or cells adopting TRIzol reagent (Thermo Fisher Scientific, Wilmington, DE, USA). Subsequently, tests of the purity and concentration of RNA were performed via exerting a NanoDrop 2000 spectrophotometer (Thermo Fisher Scientific), and synthesis of a complementary DNA (cDNA) was via adopting the Universal cDNA Synthesis Kit (Roche, Basel, Switzerland) in the light of the method of the kit. RT-qPCR was implemented on the ABI 7500 real-time PCR system (Applied Biosystems, Foster City, California, USA) exerting Power SYBR Green Master Mix (located in Takara, Dalian, China). The standardization of adoption of Β-ACTIN and U6 was for the messenger RNA (mRNA) and miRNA, respectively. Calculation of the relative expressions of miRNA and mRNA was on the grounds of the method of 2^−ΔΔCt^ [30]. The primer sequence is manifested in Table 1.

**Table 1.** RT-qPCR primer sequences

	Primer sequences (5′ – 3′)
β-actin	F: 5’- CTCCATCCTGGCCTCGCTGT-3’
	R: 5’-GCTGTCACCTTCACCGTTCC-3’
U6	F: 5’-CTCGCTTCGGCAGCACATATACT-3’
	R: 5’-ACGCTTCACGAATTTGCGTGTC-3’
MiR-21-5p	F: 5’- ACACTCCAGCTGGGTAGCTTATCAGACTGA-3’
	R:5-’CTCAACTGGTGTCGTGGAGTCGGCAATTCAGTTGAGTCAACATC-3’
KANSL2	F: 5’-TCCCAATGCTGCCCCAAAGCC-3’
	R: 5’- GGTTCCTGCTCCCCATCACTCCA −3’

Note: F, forward; R, reverse.

### 2.9 Western blot

The Western blot was implemented as previously described [31]. After extracting total protein from tissues and cells with Radio-Immunoprecipitation assay lysis buffer (Beyotime, Shanghai, China), sodium dodecyl sulfate polyacrylamide gel electrophoresis was implemented, and then electroblot of the protein was onto polyvinylidene fluoride membrane (Millipore, Billerica, MA, USA). Block of the membrane was with 5% skim milk, and then incubation was with the following primary antibodies: KANSL2 (1:1000, HPA038497, MilliporeSiGBMa) and β-actin (1:1000; ab181602, Abcam). Subsequently, the incubation of the membrane was with horseradish peroxidase-labeled secondary antibodies (1:2000; Beyotime). Visualization of the protein was via exerting an electrochemiluminescence kit (Promega, Madison, Wisconsin, USA).

### 2.10 The luciferase activity assay

Insertion of the KANSL2 3ʹuntranslated region fragment covering the miR-21-5p target site was into the pmirGlO dual-luciferase miRNA target vector (Promega), and then into the KANSL2-wild type (WT) reporter vector. Construction of the KANSL2 mutant (MUT) reporter vector covering mutation-binding sites was conducted. Co-transfection of WT/MUT reporter gene and miR-493-5p mimic or negative control miRNA was into U87, U373, and U251 in the cells exerting LipofectamineTM 2000 (Invitrogen). After 48 h, inspection of the luciferase activity was via adopting the luciferase assay kit (Promega), and adoption of Renilla luciferase activity was as a control.

### 2.11 Data analysis

Manifestation of the data was in mean ± standard deviation (SD) (n = 3). Determination of the statistical significance of the two-group differences was via exerting Student’s t-test, and analysis and manifestation of the data were via exerting Prism software 8.0 (GraphPad Software, USA). * *P* < 0.05 was considered to be statistically dramatic. Calculation of the significance of each group was via adopting one-way analysis of variance, and correction of the variance was via exerting Tukey’s test.

## 3 Results

### 3.1 Rop restrains the proliferation, migration, and invasion of GBM

**Figure 1.**
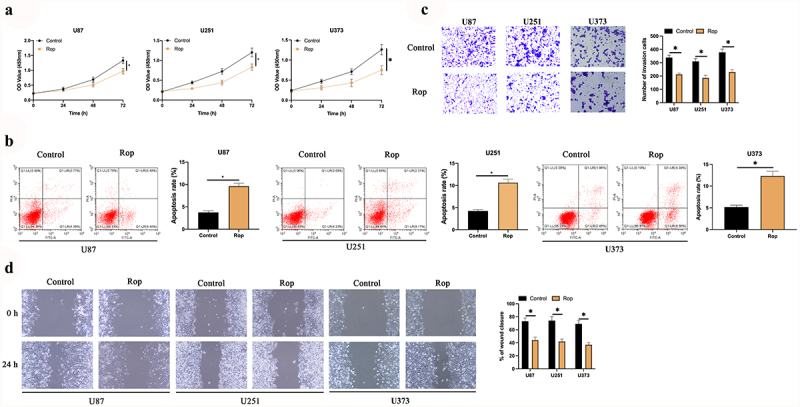
**Rop restrains the proliferation, migration and invasion of GBM**. A: CCK-8 detection of the effects of Rop treatment at different concentrations and time on GBM cell viability; B: CCK-8 test of U87, U373, and U251 cell proliferation; C: Flow cytometry examination U87, U373, and U251 cell apoptosis; D: Transwell test of U87, U373, and U251 cell invasion; E: Cell scratches examination of U87, U373, and U251 cell migration; Manifestation of values was in mean ± SD (N = 3).

To explore the impact of Rop on GBM, GBM cells were treated with different concentrations of Rop. As presented in Figure 1(a), the inhibitory effect of Rop on the cell viability of three GBM cell lines was dose- and time-dependent. Since 5 μmol/L Rop had the highest inhibitory effect, 5 μmol/L Rop was selected for subsequent experiments. The results manifested that Rop distinctly repressed the proliferation of U87 and U251 cells (Figure 1(b)). Additionally, cell apoptosis was examined via flow cytometry and Hoechst. As presented in Figure 1(c), the apoptosis rates of U87 and U251 cells were distinctly increased after Rop treatment. Subsequently, cell invasion and migration were examined via Transwell and cell scratch assay. As presented in Figure 1(d-e), the invasion and migration of U87 and U251 cells were distinctly constrained after Rop treatment. These results clarify that Rop can distinctly restrain the proliferation, invasion, and migration of GBM but boost apoptosis.

### 3.2 Augmented miR-21-5p restrains the proliferation, migration, and invasion of GBM

**Figure 2.**
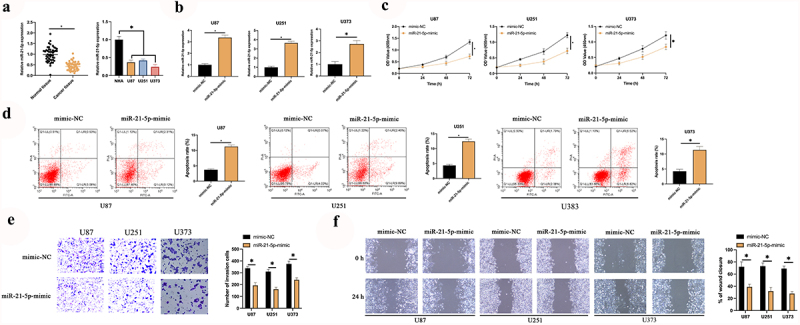
**MiR-21-5p represses the proliferation, migration and invasion of GBM**. A: RT-qPCR examination of miR-21-5p in tumor tissues, para-cancerous normal tissues, GBM U87, U373, and U251 cells, and human astrocytes NHA; B: RT-qPCR inspection of miR-21-5p in U87, U373, and U251 after transfection of miR-21-5p-mimic; C: CCK-8 test of U87, U373, and U251 cell proliferation; D: Flow cytometry examination of U87, U373, and U251 cell apoptosis; E: Transwell test of U87, U373, and U251 cell invasion; F: Cell scratches inspection of U87, U373, and U251 cell migration; C-F, the impact of transfection of miR-21-5p-mimic on them. Manifestation of values was in mean ± SD (N = 3).

The latent mechanism of Rop in restraining GBM is figured out. MiR-21-5p has been testified to be a tumor suppressor gene with decline in multiple cancers. A speculation that miR-21-5p was provided with the analogous action in GBM is manifested. Consequently, an inspection of miR-21-5p in GBM patients and cell lines was conducted. As presented in Figure 2(a), miR-21-5p was declined in GBM patients and U87, U373, and U251 cells. Additionally, miR-21-5p was distinctly linked with tumor size and grade of GBM (Table 2). Subsequently, miR-21-5p in U87, U373, and U251 cells was elevated (Figure 2(b)), and inspection of the influence of elevated miR-21-5p on the biological progression of GBM was conducted. As presented in Figure 2(c-d), after elevating miR-21-5p, U87, U373, and U251 cell proliferation was critically repressed, but cell apoptosis rate was distinctly augmented. Additionally, the overexpression of miR-21-5p also reduced the invasion and migration ability of U87 and U251 cells (Figure 2(e-f)). The results manifested miR-21-5p was declined in GBM, and elevated miR-21-5p was available to constrain GBM’s growth.

**Table 2.** Association of miR-21-5p and clinicopathological characteristics of patients in human glioma cells

Characteristic	Groups	Cases	MiR-21-5p		*P*
			The elevated (n = 24)	The declined (n = 25)	
Age (years)	< 45	30	17	13	0.1762
	≥ 45	19	7	12	
Gender	Male	21	10	11	0.8690
	Female	28	14	14	
Tumor size (cm)	< 3	31	21	10	0.0006*
	≥ 3	18	3	15	
Family history of cancer	Yes	16	6	10	0.2630
	No	33	18	15	
WHO’s Grade	I + II	11	2	9	0.0203*
	III + IV	38	22	16

### 3.3 MiR-21-5p exerts the crucial action in Rop repressing GBM’S growth

**Figure 3.**
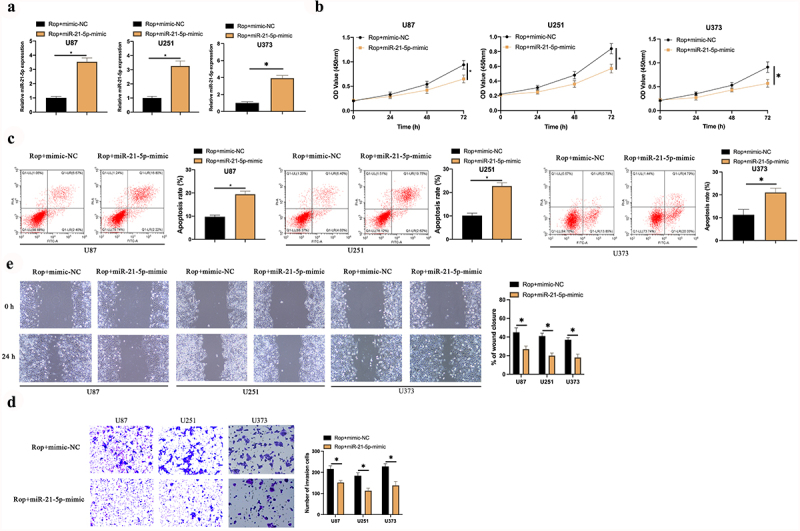
**MiR-21-5p exerts the crucial action in Rop suppressing GBM’s growth**. A: RT-qPCR test of miR-21-5p in Rop-treated U87, U373, and U251 cells after transfection of miR-21-5p-mimic; B: CCK-8 examination of Rop-treated U87, U373, and U251 cell proliferation; C: Flow cytometry test of Rop-treated U87, U373, and U251 cell apoptosis; D: Transwell examines Rop-treated U87, U373, and U251 cell invasion; E: Cell scratch examination of Rop-treated U87, U373, and U251 cell migration; B-E, the influence of transfection of miR-21-5p-mimic on them. Manifestation of values was in mean ± SD (N = 3).

For examination of whether miR-21-5p implicated in Rop restraining GBM’S growth, miR-21-5p-mimic was transfected in Rop-treated U87, U373, and U251 cells (Figure 3(a)). It turned out (Figure 3(b-c)) the proliferation ability of the U87, U373, and U251 cell was lower, but its apoptosis rate was higher in the Rop + miR-21-5p-mimic vs. the Rop + mimic-NC. Additionally, the invasion and migration abilities of U87 and U251 cells in the Rop + miR-21-5p-mimic were distinctly lower than those in the Rop + mimic-NC (Figure 3(d-e)). In brief, miR-21-5p was the critical gene in Rop constraining GBM.

### 3.4 KANSL2 is the target gene of miR-21-5p

**Figure 4.**
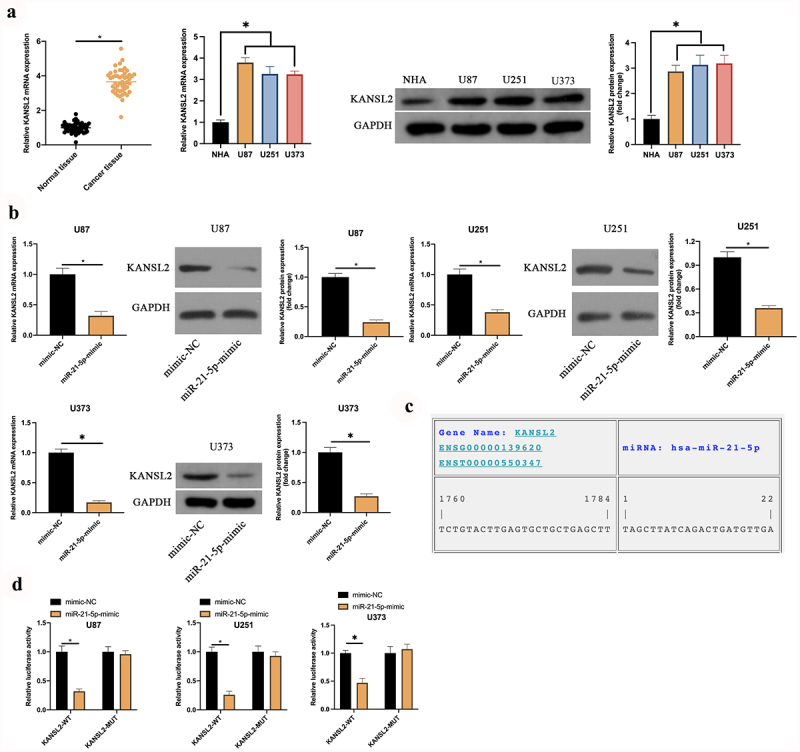
**KANSL2 is the target gene of miR-21-5p**. A: RT-qPCR and Western blot detection of KANSL2 in tumor tissues, para-cancerous normal tissues, GBM U87, U373, and U251 cells, and human astrocytes NHA; B: RT-qPCR and Western blot test of KANSL2 in U87, U373, and U251 cells after elevating miR-21-5p; C: https://cm.jefferson.edu/ website prediction of the latent-binding sites of miR-21-5p with KANSL2; D: The luciferase activity assay verification of the targeting of miR-21-5p with KANSL2. Manifestation of values was in mean ± SD (N = 3).

The downstream target genes of miR-21-5p were explored. MiR-21-5p frequently exerted the action in diseases via modulating downstream target genes. Foregoing research studies have elaborated KANSL2 is elevated in GBM and mediates cell self-renewal [20]. It is consistent with this research (Figure 4(a)). In order to explore whether miR-21-5p modulated KANSL2, the impact of elevated miR-21-5p on KANSL2 was examined. As manifested in Figure 4(b), after augmenting miR-21-5p, KANSL2 in U87, U373, and U251 cells was distinctly declined. Consequently, it has been speculated that KANSL2 is the latent target gene of miR-21-5p. Subsequently, through the prediction of the website https://cm.jefferson.edu/, it was discovered that miR-21-5p and KANSL2 had latent-binding sites (Figure 4(c)). Additionally, the targeting association between miR-21-5p and KANSL2 was further verified. As manifested in Figure 4(d), WT KANSL2 can distinctly reduce the luciferase activity of the miR-21-5p-mimic, while the MUT one exerted no distinctive influence on it. To sum up KANSL2 was the target gene of miR-21-5p in GBM.

### 3.5 KANSL2 participates in Rop constraining GBM

**Figure 5.**
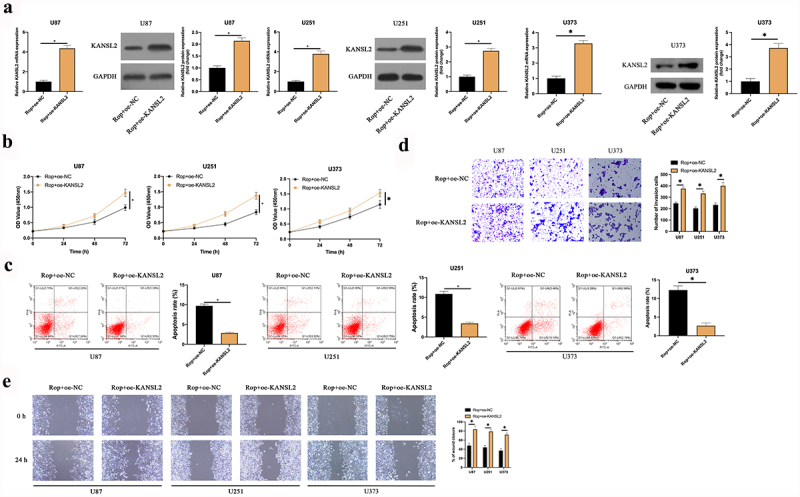
**KANSL2 implicates in the process of Rop repressing GBM**. A: RT-qPCR and Western blot test of KANSL2 in Rop-treated U87, U373, and U251 cells after transfection of oe-KANSL2; B: CCK-8 examination of Rop-treated U87, U373, and U251 cell proliferation; C: Flow cytometry examination of Rop-treated U87, U373, and U251 cell apoptosis; D: Transwell examination of Rop-treated U87, U373, and U251 cell invasion; E: Cell scratch inspection of Rop-treated U87, U373, and U251 cell migration; B-E, the influence of transfection of oe-KANSL2 on them. Manifestation of values was in mean ± SD (N = 3).

To explore whether KANSL2 was implicated in Rop repressing GBM, oe-KANSL2 was transfected into Rop-treated U87, U373, and U251 cells (Figure 5(a)). It turned out (Figure 5(b-c)) compared with the Rop + oe-NC, the proliferation of the U87, U373, and U251 cell was critically increased, and the apoptosis was distinctly reduced in the Rop + oe-KANSL2. Additionally, the overexpression of KANSL2 also distinctly increased the invasion and migration ability of the Rop-treated cells (Figure 5(d-e)). In short, KANSL2 participated in Rop restraining GBM.

### 3.6 Rop constrains GBM’s growth via modulating the miR-21-5p/KANSL2 axis

**Figure 6.**
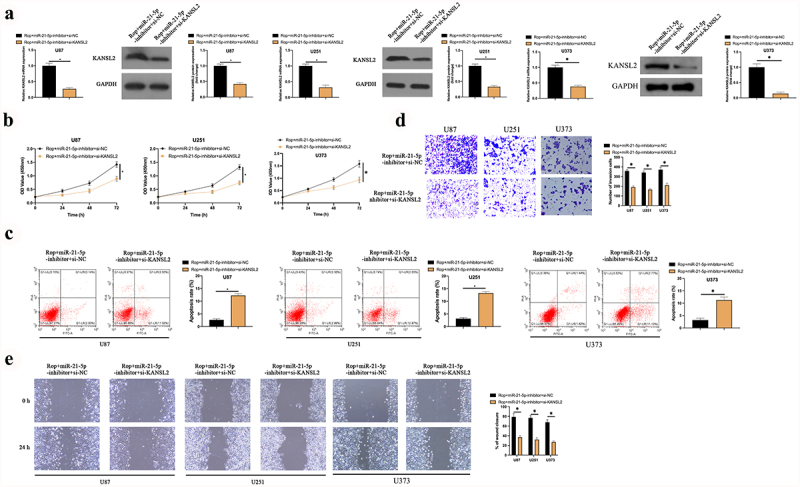
**Rop constrains GBM’s growth via mediating the miR-21-5p/KANSL2 axis**. A: RT-qPCR and Western blot tests of KANSL2 in Rop-treated U87, U373, and U251 cells; B: CCK-8 examination of Rop-treated U87, U373, and U251 cell proliferation treated with Rop; C: Flow cytometry test of Rop-treated U87, U373, and U251 cell apoptosis; D: Transwell examination of Rop-treated U87, U373, and U251 cell invasion; E: Cell scratch inspection of Rop-treated U87, U373, and U251 cell migration; A-E, the influence of co-transfection of miR-21-5p-inhibitor and si-KANSL2 on them. Manifestation of values was in mean ± SD (N = 3).

MiR-21-5p-inhbitor and si-KANSL2 was co-transfected into Rop-treated U87, U373, and U251 cells to figure out whether the miR-21-5p/KANSL2 axis was the crucial target for Rop-repressing GBM growth. As manifested in Figure 6(a), after co-transfection with si-KANSL2, KANSL2 in U87, U373, and U251 cells was distinctly declined. Additionally, after transfection of miR-21-5p-inhibitor, proliferation, migration, and invasion of U87, U373, and U251 cell were distinctly augmented, and the apoptosis was distinctly reduced. After co-transfection with si-KANSL2, these actions were distinctly reversed (Figure 6(b-e)). In short, Rop repressed proliferation, migration, and invasion of the GBM but boosted apoptosis via modulating the miR-21-5p/KANSL2 axis.

## 4 Discussion

As numerous drugs are adopted in the cure of GBM, exploring the latent mechanism of GBM drugs exerts a crucial role in clinical applications [32]. In this research, Rop treatment was made available to distinctly restrain the biological process of GBM cells in *vitro*. Additionally, Rop impacted the GBM cell growth via mediating the miR-21-5p/KANSL2 signaling pathway. It is of critical significance for seeking novel therapeutic targets.

Recently, the application of local anesthetics to cancer therapy has been discovered to be an imperative measure [33,34]. For instance, Rop constrains the proliferation and migration of colorectal cancer via silencing Integrin beta-1 and influencing its downstream pathways [35]. Rop boosts liver cancer cell apoptosis via disrupting mitochondrial function and the caspase-3 pathway’s activation [36]. Additionally, Rop is available to stimulate oxidative stress and cell apoptosis of gliomas and repress cancer cell proliferation via controlling the circular RNA (circ) SCAF11/miR-145-5p axis [37]. The present research has manifested that Rop represses cervical cancer cell growth via declining miR-96 to modulate the MEG2/pSTAT3 axis [38]. In this research, Rop was available to constrain GBM cell proliferation, invasion, and migration and boost apoptosis, illuminating that Rop was provided with a superior latent in preventing neurological tumors’ deterioration. Additionally, this study discovered 5 μmol/L Rop had the best inhibitory effect on GBM cell viability in *vitro*. Nevertheless, the dosage of Rop in clinical treatment needs to be further explored. It is worth noting that numerous studies have affirmed that Rop as a neuroanesthetic can damage neurons [39,40]. Consequently, the use of Rop in the clinical treatment of GBM might damage normal surrounding neurons.

The action of miRNA in tumors has been extensively reported. Numerous researches have elucidated miRNAs exerts a role as a regulator in GBM’s pathogenesis [41]. Presently, Yu K *et al*. have clarified that 309 miRNAs are maladjusted in GBM tissues, and MIR155HG/miR-129-5p/complement Component 1ʹs is the latent marker and cure target for GBM [42]. Additionally, Luo C *et al*. have maintained miR-640 boosts GBM cell proliferation and adhesion via targeting SLIT1 [43]. Studies have also illuminated circPARP4 accelerates GBM’s progress via sponging fucosyltransferase 4 [44]. Foregoing studies have elaborated miR-21-5p is aberrant in GBM extracellular vesicles [45] and is available to negatively mediate p21 in the p53 network [46]. Meanwhile, in this research, augmented miR-21-5p is available to constrain the proliferation, migration, and invasion of GBM but boost apoptosis, which is consistent with foregoing studies [47]. Additionally, elevated miR-21-5p is available to further strengthen Rop’s suppression on GBM.

KAT8 of KANSL2 gene encoding, a member of the KANSL protein family, modulates the NSL complex subunit 2 protein [48] belonging to the KAT8/MOF-NSL complex [47], and it has been discovered to be associated with cancer [49] and neurodevelopmental disorders [50]. Antecedent researches have elucidated that KANSL2 is elevated in GBM, which accelerates tumorigenesis via mediating GBM’s cancer stem cell-like characteristics [51]. Additionally, the discovery that KANSL2 modulates cholangiocarcinoma cell invasion is manifested. In this research, elevated KANSL2 was made available to turn around Rop’s suppression on GBM, and miR-21-5p targeted KANSL2 on GBM. It turned out KANSL2 was the crucial protein for Rop to constrain GBM. Furthermore, the results of this research intensified KANSL2ʹs cognition as a GBM proto-oncogene. Nevertheless, it is of necessity to further verify Rop’s action in mediating miR-21-5p/KANSL2 in animal models and clinics. Additionally, further determination of the molecular targets and latent pathways modulated via KANSL2 in GBM should be implemented.

## 5 Conclusion

In brief, Rop restrained the proliferation, invasion, and migration of GBM, but boosted apoptosis in *vitro* via mediating the miR-21-5p/KANSL2 pathway. The results illuminated and replenished the latent molecular mechanism of Rop modulating GBM’s biological process, offering novel data support for Rop’s clinical application as a drug to cure GBM.

## Supplementary Material

Supplemental Material
