# Supplementary figures and images for: Ropivacaine represses the proliferation, invasion, and migration of glioblastoma via modulating the microRNA-21-5p/KAT8 regulatory NSL complex subunit 2 axis
Source: Bioengineered. 2022 Feb 22;13(3):5975–86. doi: 10.1080/21655979.2022.2037955 (PMC8973733; doi:10.1080/21655979.2022.2037955)

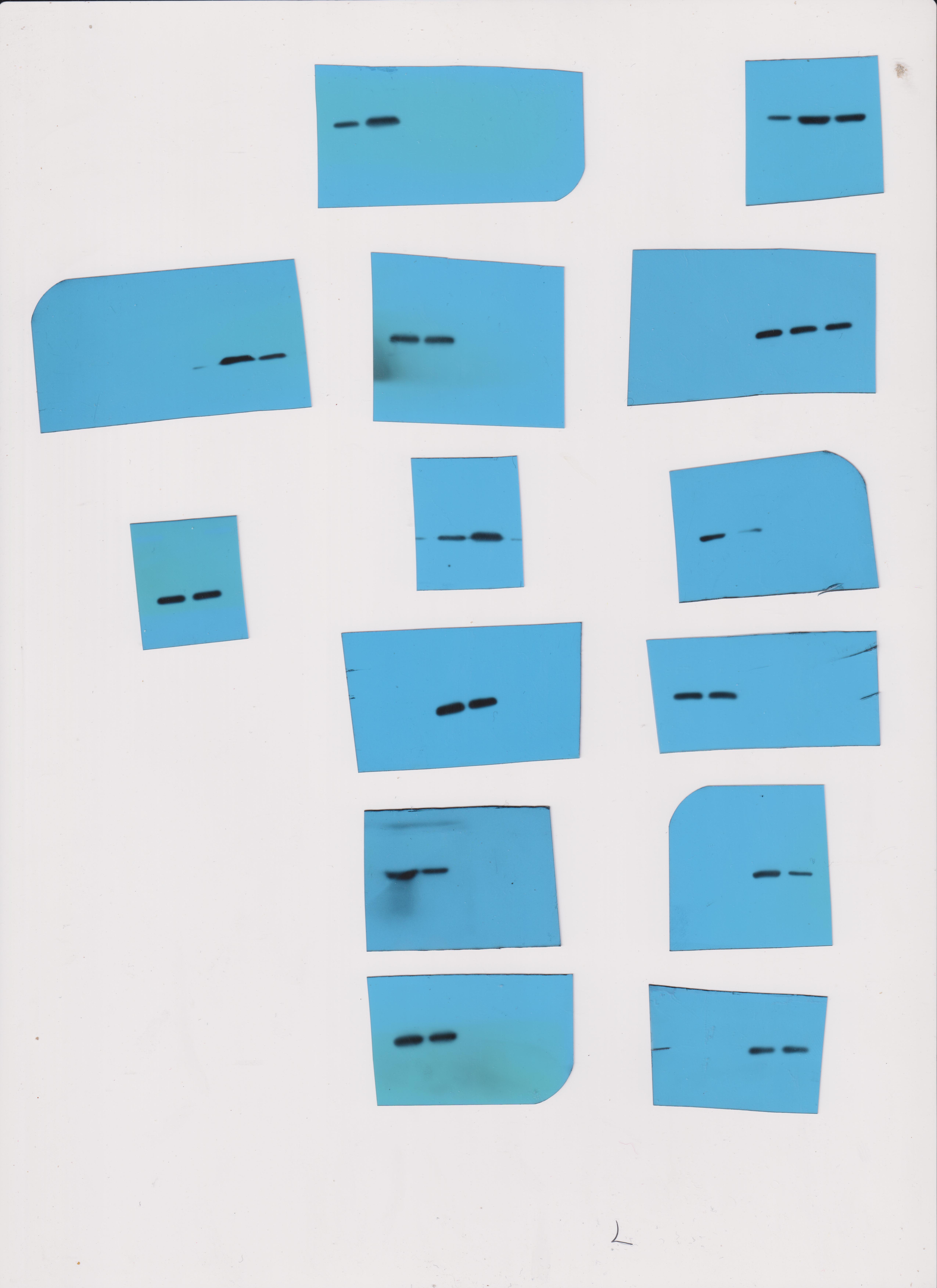

Supplement: Supplemental Material [file KBIE_A_2037955_SM7619.jpeg]
